# Development of Periodontal Organoid-like Constructs Using Human Periodontal Ligament and Gingival Epithelial Cells: Comparison of Two Assembly Strategies

**DOI:** 10.3390/gels12080692

**Published:** 2026-08-03

**Authors:** Luiza de Oliveira Matos, Mariane Beatriz Sordi, Anahid Ahmadi Birjandi, Paul Thomas Sharpe, Ariadne Cristiane Cabral Cruz

**Affiliations:** 1 Centre for Craniofacial and Regenerative Biology, Faculty of Dentistry, Oral & Craniofacial Sciences, Guy’s Hospital, King’s College London, London SE1 9RT, UK; matosluizaa@gmail.com (L.d.O.M.); marianesordi@hotmail.com (M.B.S.); anahid.ahmadi_birjandi@kcl.ac.uk (A.A.B.); paul.sharpe@kcl.ac.uk (P.T.S.); 2 Centre for Dental Implants Research, Federal University of Santa Catarina, Florianópolis 88040-900, SC, Brazil; 3 Applied Virology Laboratory, Federal University of Santa Catarina, Florianópolis 88040-900, SC, Brazil

**Keywords:** periodontal organoids, periodontal ligament cells, human gingival epithelial cells, regeneration, three-dimensional cell culture

## Abstract

Periodontal organoid research remains underdeveloped, largely due to the lack of standardized fabrication protocols and multicellular constructs capable of reproducing epithelial–mesenchymal interactions relevant to periodontal biology. In particular, it remains unclear whether different spatial assembly strategies influence construct stability, multicellular organization, or early molecular behavior in periodontal three-dimensional (3D) systems. Therefore, this study aimed to develop and compare two different methods for generating dual-lineage 3D periodontal organoid-like constructs using human periodontal ligament cells (hPDL) and human gingival epithelial cells (hGEP). These strategies were selected to compare two biologically and technically distinct spatial configurations: bilayer assembly to partially mimic epithelial–connective tissue compartmentalization, and surface seeding as a simplified fabrication approach with potential advantages for reproducibility and workflow standardization. Both cell types were cultured in different media conditions (CnT-57, DMEM, and a 1:1 CnT-57/DMEM mixture) to determine compatibility for co-culture applications. Cell viability was assessed on days 1, 3, and 7 using the MTS assay. Constructs were produced in hyaluronic acid-based hydrogels using two strategies: Group 1–sequential photopolymerization of hPDL and hGEP layers to generate a bilayer construct; and Group 2—encapsulation of hPDL followed by direct seeding of hGEP onto the construct surface. Viability within constructs was evaluated on days 3 and 7 using the Live/Dead assay. Morphology was monitored using stereomicroscopy on days 0, 1, 3, and 7. Exploratory RNA sequencing was performed on day 7 to characterize transcriptomic profiles. All tested culture media maintained cellular viabilities above 70%, with no statistically significant differences among conditions (*p* > 0.05), indicating biocompatibility for both cell types. Group 1 exhibited viabilities of 86.54% ± 8.55% and 90.69% ± 7.88% on days 3 and 7, respectively, while Group 2 showed viabilities of 87.00% ± 9.58% and 88.08% ± 9.12%, with no significant intergroup differences (*p* > 0.05). Morphological analyses demonstrated preservation of construct integrity and progressive interaction between epithelial and mesenchymal compartments. Exploratory RNA sequencing revealed only subtle transcriptomic differences between assembly strategies. In conclusion, both methodologies successfully generated viable and structurally stable dual-lineage periodontal organoid-like constructs within a hyaluronic acid-based matrix. Comparison of these two assembly strategies demonstrates the feasibility of generating reproducible multicellular periodontal 3D models using distinct spatial configurations, establishing a proof-of-concept platform for future optimization toward periodontal disease modeling, regenerative studies, and advanced biofabrication applications.

## 1. Introduction

Cell culture techniques are fundamental tools for investigating cellular functions, behaviours, and responses to different stimuli under controlled conditions [1]. Widely applied in medicine and biotechnology, two-dimensional (2D) cell culture has long served as a reference method for studies in drug development, tissue engineering, and disease modelling [2]. In this approach, cells are seeded on rigid plastic substrates, where they grow as a monolayer [3,4]. However, over time, it has become evident that 2D culture systems fail to accurately reproduce the complex physiological environment experienced by cells within living tissues [1].

Because cells in vivo interact within a three-dimensional (3D) extracellular matrix (ECM), 3D culture systems have emerged as more physiologically relevant models for studying cellular physiology and disease mechanisms. Among these, organoids, defined as 3D multicellular structures derived from stem cells or primary cells, stand out for their ability to self-organize, self-renew, and differentiate, mimicking key aspects of the architecture and function of their tissue of origin [5]. Organoid models have shown remarkable potential in applications such as drug screening, disease modelling, regenerative medicine, and personalized therapies [6,7].

In medicine, organoid technology has advanced significantly, with well-established models for organs such as the intestine [8,9], liver [10,11,12], and lung [13,14]. In contrast, periodontal organoid research remains in its early stages, with relatively few studies focused on reproducing the multicellular complexity of periodontal tissues. The periodontium is a highly specialized and dynamic tissue complex composed of gingival epithelium, periodontal ligament, cementum, alveolar bone, vascular networks, immune cells, and extracellular matrix components, all of which interact to maintain tissue homeostasis and mechanical stability [15]. This structural complexity represents a major challenge for in vitro modeling. Current periodontal 3D models are generally limited to monocultures or simplified co-culture systems that do not adequately reproduce the epithelial–mesenchymal interactions essential for periodontal physiology, disease progression, and regeneration [15]. These limitations restrict the translational relevance of available models for investigating periodontitis, host–pathogen interactions, inflammatory responses, and regenerative therapies. Given the increasing demand for physiologically relevant in vitro systems capable of reducing animal use while improving biological predictability, the development of reproducible periodontal 3D models has become highly relevant for both disease modeling and regenerative research.

No consensus currently exists regarding how to reproducibly assemble multicellular periodontal constructs that preserve epithelial–mesenchymal organization while remaining experimentally feasible and biologically stable. The development of periodontal organoid-like systems presents several challenges, including optimization of scaffold materials capable of mimicking the ECM, selection of biologically relevant cell populations, and standardization of fabrication protocols [15]. Among scaffold materials, hyaluronic acid (HA) is particularly attractive due to its established role in periodontal wound healing, hydration maintenance, ECM mimicry, and support of cell migration and proliferation [15].

Human periodontal ligament cells (hPDL) were selected for this study due to their well-documented mesenchymal stem/progenitor-like characteristics, including clonogenicity, self-renewal capacity, and multilineage differentiation potential, in addition to their essential role in ECM remodeling, mechanotransduction, and periodontal regeneration [15]. Human gingival epithelial progenitor cells (hGEP) were incorporated to represent the epithelial compartment, as these cells display proliferative and differentiation potential relevant to epithelial maintenance, barrier function, and epithelial–mesenchymal signaling [15]. The interaction between hPDL and hGEP is particularly relevant because epithelial–mesenchymal crosstalk regulates key periodontal processes, including ECM homeostasis, wound healing, inflammatory signaling, barrier maintenance, and tissue regeneration [16,17]. Therefore, incorporating both cell populations may better reproduce the structural and signaling dynamics of native periodontal tissues in 3D constructs [15].

Although several studies have explored periodontal 3D culture systems, few have investigated dual-lineage human-derived periodontal constructs integrating epithelial and periodontal ligament cell populations within a biomimetic hydrogel environment [15]. Furthermore, there is currently no standardized protocol for assembling such multicellular constructs, and it remains unclear whether different assembly strategies influence not only biological organization but also practical aspects such as reproducibility and construct fabrication workflow. However, whether different assembly strategies can influence multicellular organization, construct reproducibility, and early molecular behavior in dual-lineage periodontal organoid-like systems remains unknown. Therefore, this proof-of-concept study aimed to develop and compare two distinct assembly strategies for generating dual-lineage 3D periodontal organoid-like constructs using hPDL and hGEP embedded in HA-based hydrogels. By comparing these two fabrication approaches, we sought to evaluate whether different initial spatial configurations influence construct viability, morphology, and early transcriptomic behavior, thereby contributing to the standardization of future periodontal 3D models for translational applications.

## 2. Results and Discussion

### 2.1. Evaluation of Culture Medium Compatibility

**Figure 1:**
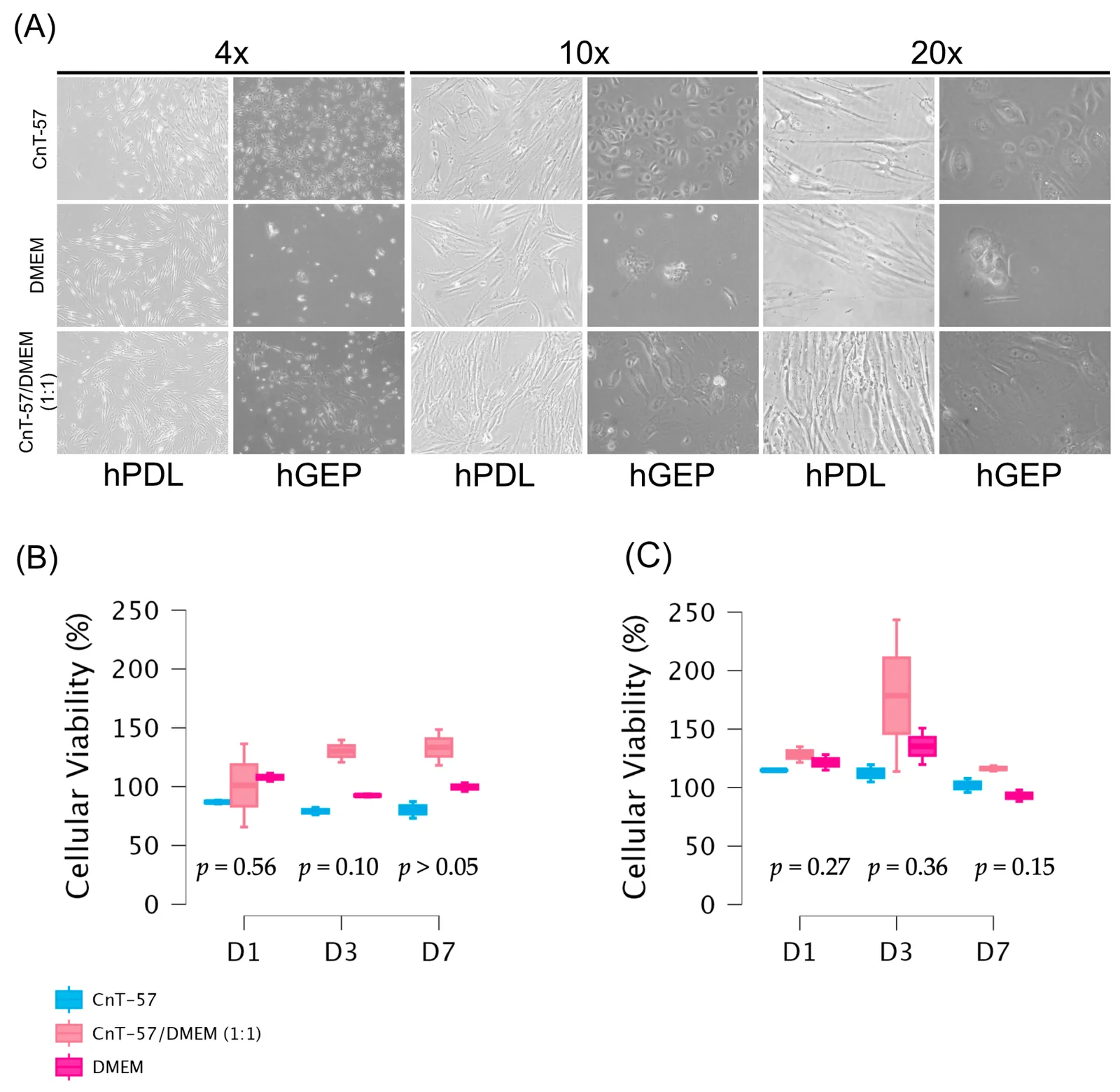
(**A**) Representative optical microscopy images of human periodontal ligament cells (hPDL) and human gingival epithelial cells (hGEP) cultured with three different culture media: CnT-57 Epithelial Proliferation Medium, Dulbecco’s Modified Eagle Medium (DMEM), and mixed medium composed of CnT-57 and DMEM (1:1), at 4×, 10×, and 20× magnifications. Images were obtained on day 3 of culture; Cellular viability of (**B**) hPDL and (**C**) hGEP. MTS colorimetric assay was employed on days 1, 3, and 7. Viability percentages were determined relative to the positive control (CnT-57 for hGEP and DMEM for hPDL).

Optical microscopy demonstrated that both hPDL and hGEP adhered and spread appropriately under all tested media conditions, including CnT-57, DMEM, and the 1:1 CnT-57/DMEM mixture (Figure 1A). No evident morphological abnormalities or signs of cytotoxicity were observed throughout the experimental period.

For hPDL, no statistically significant differences were detected among media conditions on days 1, 3, or 7 (*p* = 0.56, *p* = 0.10, *p* > 0.05, respectively). Similarly, no significant differences were observed among media conditions for hGEP on days 1, 3, or 7 (*p* = 0.27, *p* = 0.36, *p* = 0.15, respectively) (Figure 1B,C).

A critical challenge in multicellular periodontal systems is the identification of culture conditions capable of simultaneously supporting epithelial and mesenchymal populations. These cell types differ substantially in metabolic requirements, growth factor dependence, and adhesion behavior, which frequently complicates co-culture standardization. In the present study, CnT-57, DMEM, and the mixed formulation all maintained viability above 70% for both cell types at all evaluated time points, consistent with ISO 10993-5:2009 [18], indicating short-term compatibility for co-culture applications. However, viability alone should not be interpreted as evidence of functional equivalence. Cellular survival under non-native conditions may reflect metabolic adaptability rather than maintenance of lineage-specific functionality. Epithelial barrier formation, ECM deposition, cytokine secretion, and lineage commitment may remain highly sensitive to medium composition despite preserved viability. Therefore, although the mixed medium appears suitable for proof-of-concept applications, future studies should investigate whether culture conditions influence long-term construct maturation and biological function.

### 2.2. Morphological Characterization of Periodontal Organoid-like Constructs

**Figure 2:**
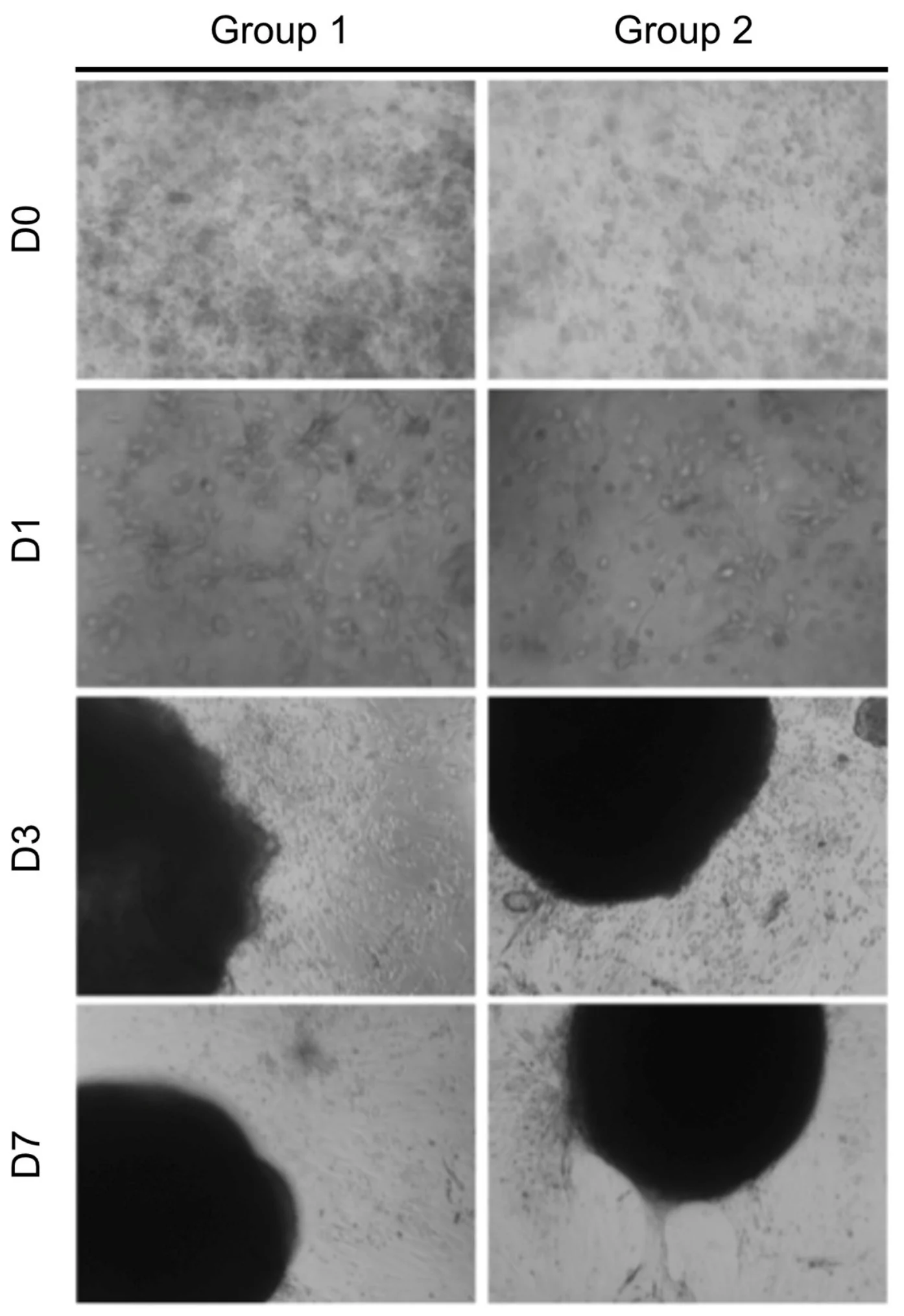
Representative stereomicroscopy images of the two organoid-like constructs on days 0, 1, 3, and 7. Columns from left to right correspond to: Group 1—separate constructs composed of human periodontal ligament cells (hPDL) and human gingival epithelial cells (hGEP) layered on top of one another; and Group 2—hPDL cells encapsulated in hyaluronic acid (HA) with hGEP seeded on the surface.

In Group 1, immediately after fabrication (day 0), two visually distinguishable compartments corresponding to the sequential assembly process were observed. Over time, constructs exhibited progressive morphological continuity at the interface between layers, particularly by days 3 and 7 (Figure 2). In Group 2, constructs initially displayed a compact central hydrogel region surrounded by cells distributed along the construct surface. By days 3 and 7, surface coverage appeared more homogeneous, while overall construct morphology remained preserved (Figure 2). Both groups maintained structural integrity and overall morphological stability throughout the experimental period, suggesting that both assembly strategies support stable 3D multicellular constructs under the tested conditions.

It should be noted that these observations are based exclusively on qualitative brightfield and stereomicroscopy analyses. Fluorescence-based lineage tracking, confocal microscopy, and z-stack reconstruction were not performed in this proof-of-concept study; therefore, definitive assessment of cell-specific spatial distribution, migration, and epithelial–mesenchymal interactions could not be established. Because lineage-specific labeling was not performed, observed changes in construct morphology cannot be directly attributed to cell migration or intercellular physical interactions.

Although several periodontal 3D models have been described, multicellular periodontal constructs integrating epithelial and mesenchymal components remain scarce. Golda et al. [19] developed an organotypic gingival model using telomerase-immortalized gingival keratinocytes (TIGKs) and immortalized gingival fibroblasts to study host–pathogen interactions. While valuable for infection studies, this model did not incorporate periodontal ligament-derived cells, thereby limiting its relevance for investigating periodontal connective tissue biology and regeneration. Similarly, Dabija-Wolter et al. [20] generated junctional and sulcular epithelium models by culturing gingival keratinocytes on matrices containing gingival or periodontal ligament fibroblasts. However, these models primarily focused on epithelial differentiation and barrier formation rather than multicellular 3D assembly or epithelial–mesenchymal structural organization. Reuther et al. [21] described a 3D co-culture model containing periodontal ligament fibroblasts and alveolar bone cells, representing an important advance in periodontal tissue engineering. Nevertheless, the absence of an epithelial compartment limits the translational applicability of this model for studying epithelial–mesenchymal crosstalk, which is fundamental to periodontal wound healing, inflammatory signaling, and tissue regeneration. The most conceptually comparable model was reported by Yamada et al. [22], who generated multicellular 3D constructs using porcine epithelial and fibroblastic cells derived from periodontal ligament. Although this study demonstrated the feasibility of multicellular periodontal assembly, the use of animal-derived cells and limited epithelial characterization reduced the translational applicability relative to human-derived systems.

Collectively, these studies demonstrate that currently available periodontal 3D models generally prioritize either epithelial differentiation, fibroblast biology, or infection modeling, but rarely integrate human epithelial and periodontal ligament populations within a unified biomaterial platform. In this context, the present study contributes by comparing two distinct assembly strategies for generating dual-lineage periodontal organoid-like constructs within a hyaluronic acid-based hydrogel.

Both assembly strategies successfully generated structurally stable constructs throughout the 7-day culture period. In Group 1, sequential bilayer fabrication initially imposed spatial segregation between cell populations, followed by progressive interface continuity over time. This architecture may partially mimic the compartmentalized organization observed in native periodontal soft tissues, where epithelial and connective compartments remain structurally distinct yet functionally interconnected. By contrast, Group 2 involved direct epithelial seeding onto pre-formed mesenchymal constructs, promoting progressive peripheral epithelial organization. These distinct architectures likely expose cells to different microenvironmental conditions, including altered matrix accessibility, nutrient diffusion, oxygen gradients, and mechanical constraints. Spatial organization is a well-established regulator of cellular behavior in 3D systems, influencing polarity, migration, matrix remodeling, and paracrine signaling [5].

Spatial organization is a well-established regulator of cellular behavior in 3D systems. Different construct architectures may alter epithelial–mesenchymal interface formation, cell–cell contact patterns, matrix accessibility, nutrient and oxygen diffusion gradients, and local mechanical constraints [5]. These microenvironmental differences can influence cell polarity, migration, cytoskeletal organization, mechanotransduction, and paracrine signaling, thereby modulating transcriptional responses [5].

### 2.3. Cellular Viability Within Constructs

**Figure 3:**
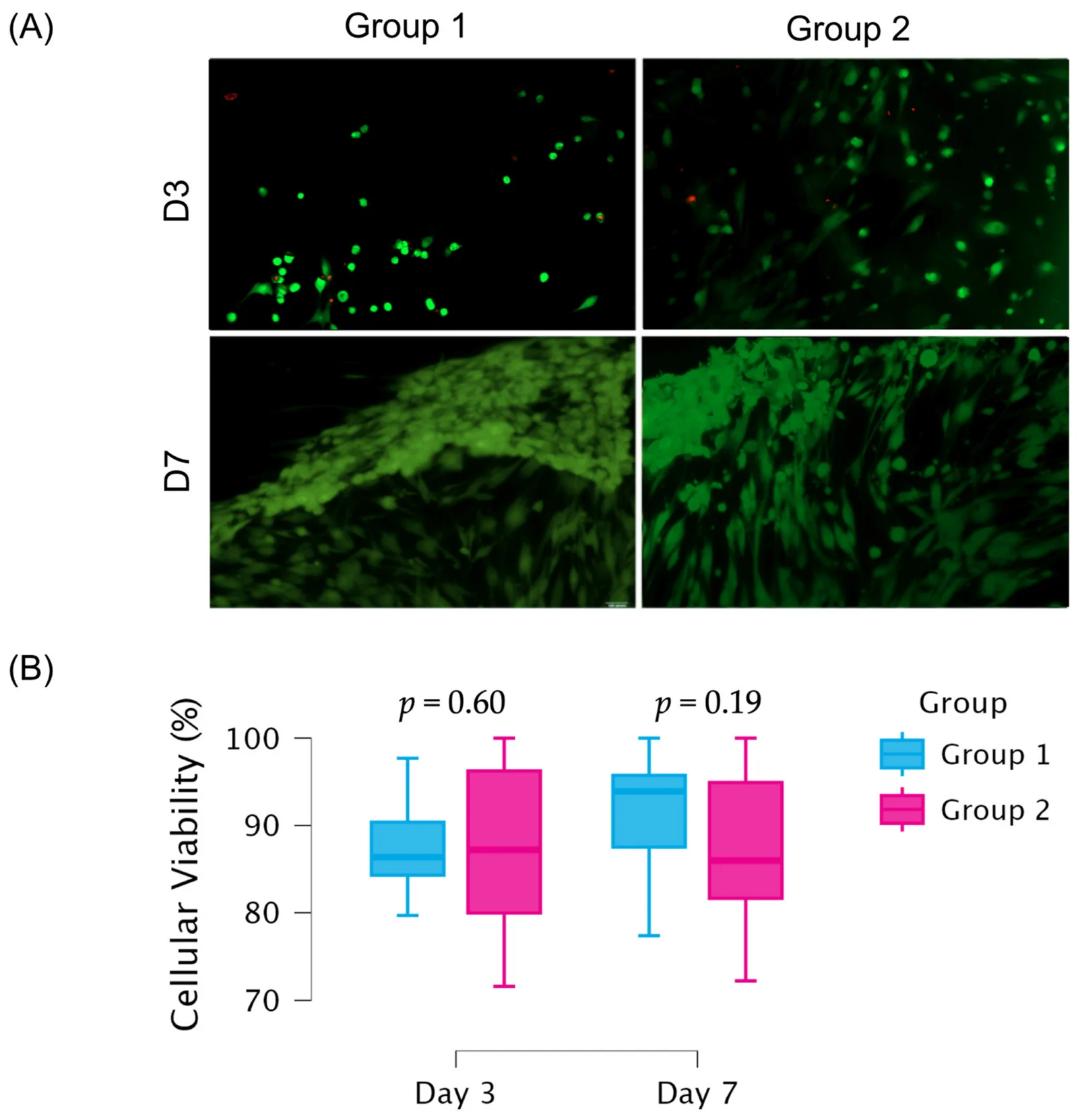
(**A**) Representative fluorescence microscopy images of Group 1 and Group 2 organoid-like constructs stained with the Live/Dead assay. Viable cells exhibited green fluorescence, whereas non-viable cells appeared in red. Scale bars: 100 µm. (**B**) Quantification of cellular viability (%) for each experimental group on days 3 and 7. Data are shown as mean ± SD from 15 images per group per time point.

Live/Dead fluorescence analysis demonstrated high cellular viability in both constructs at all evaluated time points (Figure 3). Group 1 exhibited mean viabilities of 86.54% ± 8.55% on day 3 and 90.69% ± 7.88% on day 7. Group 2 showed viabilities of 87.00% ± 9.58% and 88.08% ± 9.12% on days 3 and 7, respectively. No statistically significant differences were detected between groups on day 3 (*p* = 0.06) and day 7 (*p* = 0.19). These results indicate that both fabrication strategies supported high global cell viability during short-term culture. Live/Dead staining provided global viability estimates and did not distinguish viability differences between central and peripheral construct regions. Another important aspect is that, although no major viability differences were detected between groups, it remains plausible that distinct long-term maturation profiles or functional phenotypes may emerge beyond the 7-day timepoint evaluated here.

Both groups demonstrated high global viability (>85%) during culture, indicating that the selected hydrogel formulation and photopolymerization parameters were biocompatible. This finding is particularly relevant because photocrosslinked hydrogels are often associated with concerns regarding photoinitiator toxicity and UV-induced cellular damage. The favorable viability observed may be partially attributed to the biological properties of HA. HA is an endogenous ECM component abundant in periodontal tissues and plays important roles in tissue hydration, viscoelasticity, wound healing, inflammatory modulation, and cell migration [23]. Furthermore, HA interacts with receptors such as CD44 and RHAMM, regulating adhesion, proliferation, cytoskeletal organization, and mechanotransduction.

These properties likely contributed not only to cell survival but also to maintenance of structural integrity and multicellular organization. Nevertheless, HA alone does not fully reproduce the biochemical and biomechanical complexity of native periodontal ECM, which contains collagen-rich fibrillar networks, proteoglycans, glycoproteins, and mineral-associated interfaces. Accordingly, future studies should investigate composite hydrogels or ECM-functionalized biomaterials capable of better reproducing periodontal matrix heterogeneity.

Although high cell viability was maintained throughout the experimental period, viability should not be interpreted as a surrogate for proliferation. The present study did not include dedicated proliferation analyses; therefore, whether different assembly strategies influence proliferative behavior within the constructs remains unknown. Future studies should incorporate proliferation-specific assays, such as Ki-67 immunostaining, EdU incorporation, or DNA quantification, to better characterize construct maturation dynamics.

### 2.4. Exploratory Transcriptomic Analysis

**Figure 4:**
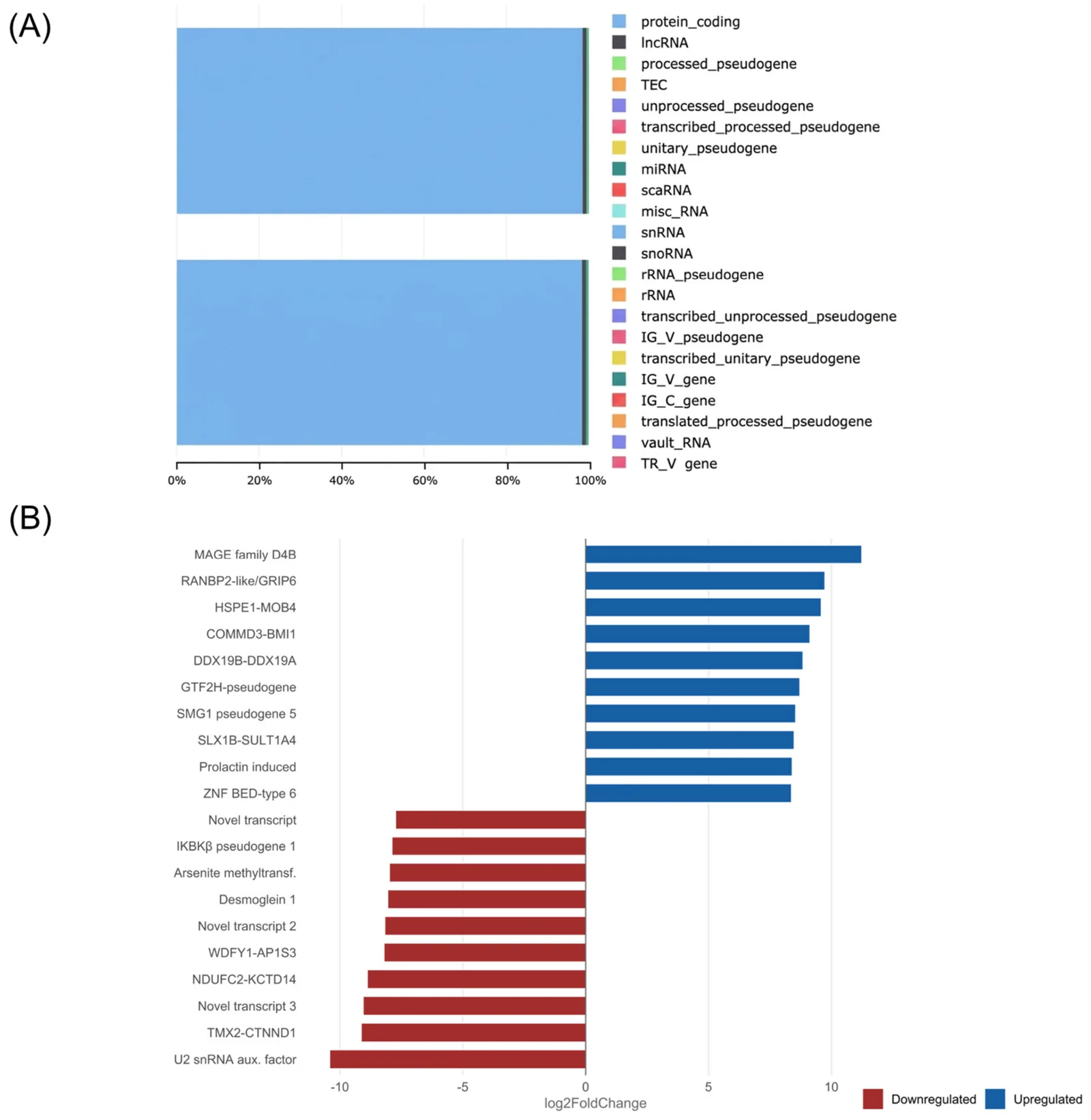
(**A**) Feature Counts biotype classification showing the relative abundance of transcript categories, with protein-coding genes representing the predominant biotype. (**B**) Representative log2 fold change in each gene.

Exploratory RNA sequencing analysis revealed overall similar transcriptomic profiles between Group 1 and Group 2 periodontal organoid-like constructs after 7 days of culture, suggesting that both assembly strategies support comparable early molecular behavior. Functional analysis (Figure 4) demonstrated only modest transcriptomic divergence between groups, without evidence of large-scale differential regulation.

Therefore, exploratory RNA sequencing revealed highly similar transcriptomic profiles between both construct groups, suggesting that short-term spatial configuration did not dramatically alter global molecular behavior. Only subtle differences in transcript abundance were identified. Genes modestly enriched in one group included transcripts associated with proliferation, intracellular trafficking, and cellular adaptation, including MAGE-D4B [24], RANBP2-like proteins [25], GRIP domain-containing 6 [26], and HSPE1-MOB4 readthrough transcripts. These genes are broadly associated with processes including cellular proliferation, intracellular trafficking, nuclear transport, and stress adaptation [24,25,26]. Conversely, relatively downregulated transcripts included genes associated with RNA processing and adhesion-related pathways, such as U2AF1-like 5 and TMX2-CTNND1 readthrough. Collectively, the overall transcriptomic similarity observed between Groups 1 and 2 suggests that both fabrication strategies generate biologically comparable periodontal organoid-like constructs under short-term culture conditions. The modest transcriptional differences identified likely reflect subtle adaptive responses to differences in initial construct geometry rather than major biological divergence between fabrication approaches. These findings support the robustness of both assembly strategies while suggesting that spatial configuration may influence early molecular adaptation in 3D periodontal systems. Given the absence of biological replicates, these transcriptomic findings should be interpreted cautiously and considered exploratory only. Future studies incorporating biological replicates and pathway enrichment analyses will be necessary to determine whether distinct construct architectures result in biologically meaningful functional differences.

Similar observations were reported by Hemeryck et al. [27], who reported configuration-dependent transcriptomic variation in epithelial organoids derived from human dental follicles cultured in Matrigel. Although their model was restricted to epithelial cells, both suggest that early spatial organization may influence molecular responses even in the absence of overt morphological differences. Importantly, transcriptomic findings from the present study must be interpreted cautiously due to the absence of biological replicates. Therefore, RNA sequencing data should be considered exploratory and hypothesis-generating only. Future studies incorporating biological replicates, pathway enrichment analyses, and cell-specific transcriptomic profiling will be necessary to determine whether different assembly strategies produce biologically distinct functional phenotypes.

A conceptual limitation of the present study concerns the classification of these constructs as organoids. Classical organoids are typically characterized by self-organization, self-renewal, multilineage differentiation, and partial functional recapitulation of native tissues. Although the constructs generated here exhibited multicellular 3D organization, epithelial–mesenchymal compartmentalization, and sustained viability within a biomimetic matrix, additional evidence of long-term maturation, lineage-specific differentiation, and tissue-specific functionality is still required. Therefore, the term periodontal organoid-like constructs more appropriately reflects the current developmental stage of this model.

Additional limitations should also be acknowledged. The 7-day culture period and inclusion of only two cell populations limit conclusions regarding long-term tissue maturation and the multicellular complexity of native periodontal tissues. Spatial organization, morphology, and viability were evaluated primarily through qualitative imaging approaches without fluorescent lineage tracing, confocal z-stack reconstruction, quantitative morphometric analyses, or region-specific viability assessment. Consequently, definitive evaluation of cell distribution, migration dynamics, epithelial–mesenchymal interface organization, and potential central necrosis within dense cellular regions was not possible. Moreover, construct morphology and viability were assessed using different imaging modalities with distinct magnification and acquisition parameters, which may influence visual interpretation of cell density and structural compactness. No direct proliferation analyses or mechanical stimulation protocols were included, despite the known importance of mechanotransduction in periodontal physiology. Finally, monoculture controls and acellular hydrogel controls were not incorporated, limiting assessment of lineage-specific behavior and isolated biomaterial effects. Future studies should incorporate longer culture periods, additional periodontal cell populations, quantitative spatial analyses, lineage-specific fluorescent labeling, confocal imaging, proliferation assays, mechanical conditioning, and appropriate control groups to enable more comprehensive functional validation of periodontal organoid-like systems.

Despite these limitations, this study establishes a reproducible proof-of-concept platform for generating dual-lineage periodontal organoid-like constructs using human-derived cells and a clinically relevant biomaterial. Future studies should incorporate longer culture periods to evaluate construct maturation and long-term stability, as well as functional assays to assess epithelial differentiation, extracellular matrix deposition, and inflammatory responsiveness. Advanced spatial characterization methods, including fluorescence-based lineage tracing, live-cell tracking, and confocal z-stack imaging, will be essential to validate cell-specific distribution, migration dynamics, and epithelial–mesenchymal interface organization throughout construct thickness. Additionally, incorporation of further periodontal cell populations, mechanical conditioning, and advanced biofabrication approaches such as 3D bioprinting, microfluidic systems, and ECM-functionalized biomaterials may enable the development of more physiologically relevant and functionally complex periodontal organoid-like models for regenerative medicine, inflammatory disease modeling, and host–microorganism interaction studies [28,29,30].

## 3. Conclusions

This study demonstrates a straightforward and reproducible approach for generating dual-lineage periodontal organoid-like constructs through the co-culture of human gingival epithelial cells and periodontal ligament fibroblasts within a hyaluronic acid-based biomimetic matrix. Both assembly methods supported the formation of structurally stable and viable three-dimensional constructs with preserved multicellular organization and only modest transcriptomic differences after 7 days of culture. These findings establish a proof-of-concept platform for the development of more sophisticated periodontal models incorporating additional cellular populations, functional analyses, mechanical stimulation, and advanced biofabrication technologies such as 3D bioprinting and microfluidic systems. Collectively, this approach may contribute to the advancement of translational research in periodontal regeneration and disease modelling, and epithelial–mesenchymal interaction studies.

## 4. Methods and Methods

### 4.1. Cell Culture

Two primary human cell types were used for the development of periodontal organoid-like constructs. Primary human gingival epithelial cells (hGEP) were originally purchased from CellnTEC Advanced Cell Systems (Bern, Switzerland) and cultured in CnT-57 epithelial proliferation medium (CellnTEC, Bern, Switzerland) [16,17]. Primary human periodontal ligament cells (hPDL) were kindly provided by Dr Ana Angelova, King’s College London. Ethical approval was obtained from the Kent United Kingdom National Health Research Ethics Committee (reference number 11/LO/0259), and all procedures were conducted in accordance with the Declaration of Helsinki. The hPDL cells were cultured with Dulbecco’s modified essential medium (DMEM, Sigma Aldrich, Darmstadt, Germany) supplemented with 10% (*v*/*v*) fetal bovine serum (FBS), 100 units/mL penicillin, and 100 μg/mL streptomycin (Gibco-Thermo Fisher Scientific, Waltham, MA, USA). Both cell types were maintained at 37 °C in a humidified atmosphere containing 95% air and 5% CO_2_. Cells from passages #2 to #5 were used in all experiments. Phosphate-buffered saline (PBS; Gibco-Thermo Fisher Scientific, Waltham, MA, USA) was employed for washing confluent cultures. Trypsin (Gibco-Thermo Fisher Scientific, Waltham, MA, USA) without phenol red was used for hGEP detachment, while conventional trypsin was used for hPDL.

### 4.2. Effect of the Culture Medium on Cellular Behaviour

Because hPDL and hGEP required distinct standard media, a compatibility assay was performed to identify conditions suitable for co-culture applications. A total of 1 × 10^5^ cells/well was plated per well in 24-well plates and cultured in one of the following media conditions: (1) CnT-57 medium; (2) DMEM; and (3) a 1:1 mixture of CnT-57 and DMEM (*v*/*v*). The same protocol was performed for both cell types. Media were replaced every 48 h. Morphological observations were conducted using an optical microscope (Olen K55-BA, Shenzhen Olen Electric Co., Shenzhen, China) at 4×, 10×, and 20× magnifications.

Cell viability was assessed using the MTS colorimetric assay (CellTiter 96^®^, Promega, Madison, WI, USA) on days 1, 3, and 7 [18]. At each time point, media were removed, cells were washed twice with PBS, and 200 µL MTS reagent was added. After 4 h of incubation, the absorbance was measured at 490 nm (CLARIOstar Plus, BMG Labtech, Ortenberg, Germany). For intra-cell-type normalization, viability values were expressed relative to the standard medium of each respective cell type (CnT-57 for hGEP and DMEM for hPDL), enabling assessment of compatibility across alternative culture conditions. Cross-cell-type comparisons were not performed using normalized data. All experiments were performed in triplicate (*n* = 3) and independently repeated three times, resulting in a total of nine observations per experimental condition (total *n* = 9).

### 4.3. Hydrogel Preparation

The hydrogel matrix was prepared using hyaluronic acid (HA) combined with sodium lithium phenyl-2,4,6-trimethylbenzoyl phosphinate (SAP) photoinitiator. SAP was prepared as a 0.025% (*w*/*v*) solution in PBS, according to manufacturer recommendations. The SAP solution was sterilized using a 0.22 μm filter. HA was then added to obtain a final concentration of 1% (*w*/*v*), based on preliminary handling optimization demonstrating cytocompatibility and appropriate viscosity for oral tissue engineering applications. The mixture was stored at 4 °C overnight to ensure complete HA hydration and gel stabilization. HA was selected due to its biological relevance in periodontal wound healing, extracellular matrix mimicry, hydration maintenance, and support of cell migration and proliferation [15].

### 4.4. Organoid Preparation

**Figure 5:**
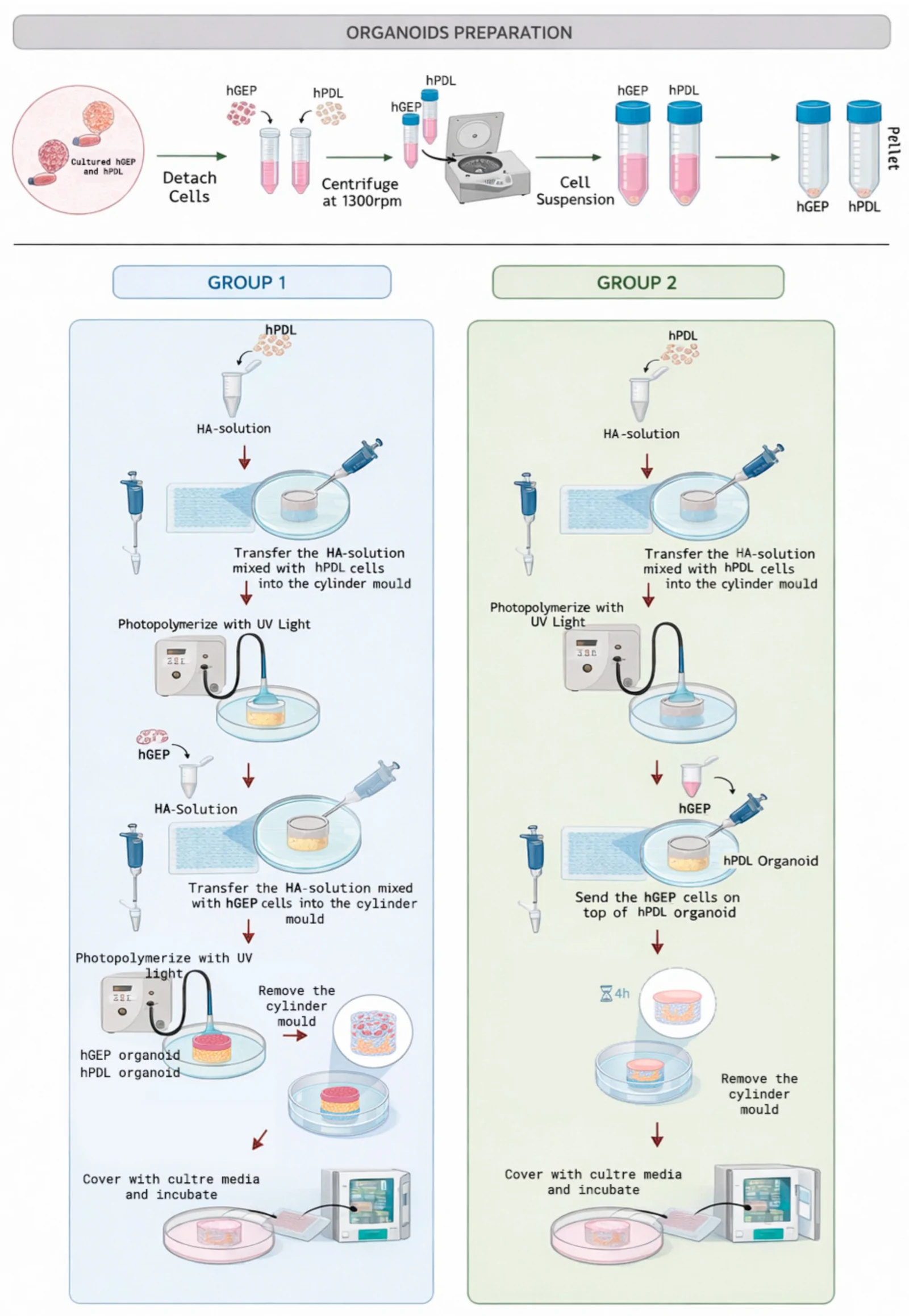
Schematic representation of periodontal organoid preparation. Human gingival epithelial progenitor cells (hGEP) and human periodontal ligament fibroblasts (hPDL) were cultured separately, detached, and centrifuged. Group 1—hPDL cells were first mixed with hyaluronic acid (HA) and photopolymerized within a cylindrical mould to form a basal layer. After polymerization, hGEP suspended in HA solution was added on top and photopolymerized again, creating a bilayer structure. The construct was demoulded, covered with culture medium, and incubated to enable interaction and maturation of the dual-lineage organoid. Group 2—hGEP cells were embedded in a HA solution and photopolymerized within a cylindrical mould, forming the inner core of the construct. After polymerization, hPDL cells were seeded around the hGEP-HA core, establishing an outer epithelial layer. The organoid was then immersed in culture medium and incubated for maturation.

Two distinct assembly strategies were evaluated (Figure 5).

Group 1 (G1): Sequential Bilayer Assembly—Following trypsinization and centrifugation (1300 rpm, 5 min), 1 × 10^5^ cells were resuspended in 40 μL HA solution, based on preliminary handling optimization. hPDL cells were first embedded in HA and transferred into cylindrical molds positioned in 48-well plates. Photopolymerization was performed using UV light (Omnicure S1000 UV Spot Curing System, EXFO, Mississauga, ON, Canada) for 1 min at the lowest available intensity. UV parameters were selected as the minimum exposure necessary to achieve construct stabilization while minimizing potential cytotoxicity. After polymerization of the hPDL basal layer, hGEP suspended in HA was added as a second layer and photopolymerized under identical conditions, resulting in a bilayer construct. Constructs were carefully demolded, covered with culture medium, and incubated under standard conditions.

Group 2 (G2): Encapsulation Plus Surface Seeding—hPDL cells were initially embedded in HA and photopolymerized as described above to form the construct core. Subsequently, hGEP cells were directly seeded onto the surface of the polymerized construct. To promote adhesion and prevent cell displacement, constructs were incubated for 4 h prior to demolding. Culture medium was then added to fully immerse constructs.

All experiments were performed in triplicate (*n* = 3) and independently repeated three times, resulting in a total of nine observations per experimental condition (total *n* = 9).

### 4.5. Morphological Analysis

Construct morphology and integrity were monitored by stereomicroscopy (SMZ1500, Nikon, Tokyo, Japan) equipped with a digital camera (Digital Sight DS-Fi1, Nikon, Tokyo, Japan) on days 0, 1, 3, and 7.

Qualitative observations focused on construct stability, layer preservation, and progressive interaction between epithelial and mesenchymal compartments. Quantitative spatial image analysis was not performed in this proof-of-concept study. All experiments were performed in triplicate (*n* = 3) and independently repeated three times, resulting in a total of nine observations per experimental condition (total *n* = 9).

### 4.6. Viability Assessment Within Constructs

Cell viability within constructs was evaluated using the Live/Dead™ Viability/Cytotoxicity Kit (Invitrogen, Waltham, MA, USA) on days 3 and 7. Constructs were incubated with staining solution containing 2 μM calcein-AM and 8 μM ethidium homodimer-1 diluted in PBS for 30 min at room temperature. Fluorescence images were acquired using an Apotome 3 fluorescence microscope (Zeiss, Jena, Germany). Images were analyzed using ImageJ 1.54r software (NIH, Bethesda, MD, USA) [31]. Viability was calculated as:

live cells/(live cells + dead cells) × 100.

This assay provided global construct viability estimates and did not distinguish regional differences between construct core and periphery.

All experiments were performed in triplicate (*n* = 3) and independently repeated three times, resulting in a total of nine observations per experimental condition (total *n* = 9).

### 4.7. RNA Sequencing

After 7 days of culture, total RNA was extracted from constructs and submitted for whole-transcriptome sequencing using the Illumina NextSeq 2000 platform (Illumina, San Diego, CA, USA), generating paired-end 100 bp reads. Raw sequencing data were processed using the nf-core/RNAseq pipeline (nf-core Community, Sweden). Adapter trimming and initial quality assessment were performed using Trim Galore and FASTQC (Babraham Bioinformatics, Cambridge, UK). Reads were aligned to the human reference genome GRCh38 (Ensembl release 113) using STAR aligner (Dobin et al., Cold Spring Harbor Laboratory, New York, NY, USA), and transcript quantification was performed using Salmon, followed by gene-level summarization with tximport.

Sequencing quality control metrics, including Phred quality scores, mapping rates, genomic origin distribution, and transcript biotype classification, were evaluated to confirm overall dataset quality and are provided in the Supplementary Materials (Figure S1). Briefly, all samples demonstrated high sequencing quality, with mean Phred scores above Q30, mapping rates above 94%, and predominant exonic read distribution, supporting dataset suitability for exploratory transcriptomic analysis. Downstream analyses were conducted in R (R Foundation for Statistical Computing, Vienna, Austria) using the DESeq2 package (Bioconductor, Seattle, WA, USA). Because biological replicates were not available, differential expression analysis was performed using an exploratory descriptive workflow only.

### 4.8. Statistical Analysis

Statistical analyses were performed using JASP software (Version 0.95.4; JASP Team, University of Amsterdam, Amsterdam, The Netherlands). Sample size determination was performed prior to the experiments based on preliminary experiments evaluating construct viability, as previously described [32]. The calculated minimum sample size was 8.36. Therefore, a total sample size of *n* = 9 was adopted in the present study. Specifically, experiments were performed using three independent samples per condition (*n* = 3), and the entire experiment was repeated three times, resulting in a final total of *n* = 9 for statistical analyses. Data normality was assessed using the Shapiro–Wilk test. Given the small sample size (*n* = 9) and non-normal distributions, non-parametric statistical methods were applied throughout. Differences among media conditions in the MTS assay were analyzed using the Kruskal–Wallis test. Comparisons between Groups 1 and 2 for Live/Dead viability were analyzed using the Mann–Whitney U test. Data are presented as mean ± standard deviation (SD). Statistical significance was set at *p* < 0.05.

## Data Availability

The data presented in this study are available on request from the corresponding author.

## Supplementary Materials

The following supporting information can be downloaded at https://www.mdpi.com/article/10.3390/gels12080692/s1: Figure S1. RNA-sequencing quality control metrics. (a) FASTQC mean Phred quality score plots demonstrating high sequencing quality across all read positions. (b) Summary table reporting STAR alignment percentages for each group. (c) QualiMap genomic origin analysis indicating that most reads mapped to exonic regions, with smaller intronic and intergenic fractions.
