# Supplementary material for: Development of Periodontal Organoid-like Constructs Using Human Periodontal Ligament and Gingival Epithelial Cells: Comparison of Two Assembly Strategies
Source: Gels. 2026 Aug 3;12(8):692. doi: 10.3390/gels12080692 (PMC13512850; doi:10.3390/gels12080692)
Supplement: Supplementary file 1 [file gels-12-00692-s001.zip › gels-4241389-supplementary.pdf]

a)

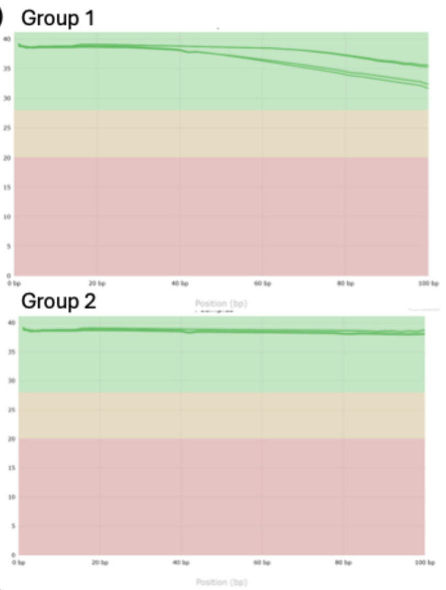

c)

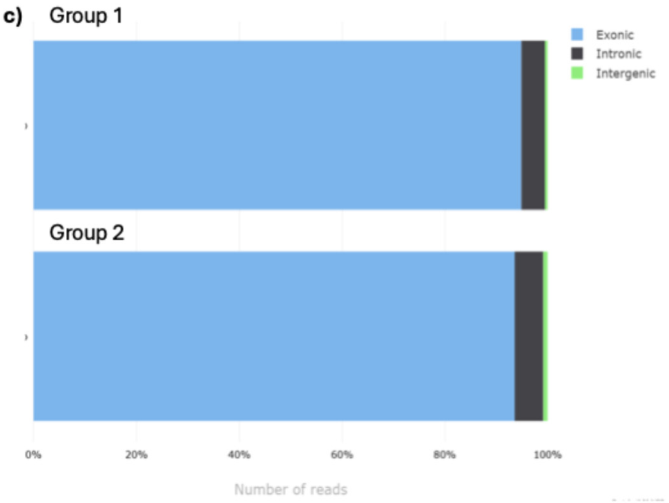

b)

Sample	Aligned reads %	Uniquely mapped reads %
Group 1	95.1	88.5
Group 2	94.8	88.5

Figure S1. RNA-sequencing quality control metrics. (**a**) FASTQC mean Phred quality score plots demonstrating high sequencing quality across all read positions. (**b**) Summary table reporting STAR alignment percentages for each group. (**c**) QualiMap genomic origin analysis in-dicating that most reads mapped to exonic regions, with smaller intronic and intergenic fractions.
